# Identification in milk of a serum amyloid A peptide chemoattractant for B lymphoblasts

**DOI:** 10.1186/1471-2172-10-4

**Published:** 2009-01-23

**Authors:** Berardo de Jesus Rodriguez, Claire Chevaleyre, Gwénaële Henry, Daniel Mollé, Isabelle Virlogeux-Payant, Mustapha Berri, François Boulay, Joëlle Léonil, François Meurens, Henri Salmon

**Affiliations:** 1 Institut National de la Recherche Agronomique (INRA), UR1282, Infectiologie Animale et Santé Publique, F-37380, Nouzilly (Tours), France; 2 UMR Science et Technologie du Lait et de l'Oeuf, INRA-Agrocampus 1253, 65 rue de Saint-Brieuc, 35042 Rennes CEDEX, France; 3 Laboratorio de Patologia Animal, Universidad de Antioquia, Medellin, Colombia; 4 Commissariat à l'Energie Atomique (CEA), DSV, iRTSV, Laboratoire Biochimie et Biophysique des Systèmes Intégrés, Grenoble, F-38054, France. CNRS, UMR 5092, 17 rue des Martyrs, Grenoble F-38054, France. Université Joseph Fourier, Grenoble, F-38000, France

## Abstract

**Background:**

Normal mammary gland contains an extravascular population of B lymphoblasts, precursors of the immunoglobulin plasma cells that play a key role in the passive protection of neonates by secreting immunoglobulins to colostrum and milk. We investigated the presence of chemoattractants in the milk by analysing the chemoattractant activity of various fractions of this secretion. Milk chemoattractants are potentially involved in the recruitment of lymphocytes from the maternal bloodstream in lactating mammary glands.

**Results:**

The dilution-related lymphoid cell chemoattraction of whey was associated with a < 10 kDa ultrafiltrate. Active fractions were purified by reverse-phase high performance liquid chromatography. Two peptides of 2.7 kDa (DMREANYKNSDKYFHARGNYDAA) and 1 kDa (RPPGLPDKY) were identified as fragments of the SAA protein family, tentatively identified as SAA2. Only the 2.7 kDa synthetic peptide displayed chemotactic activity, at two different optimal concentrations. At the lower concentration (3.7 nM), it attracted B-cell lymphoblasts, whereas at the higher (3.7 μM), it attracted B lymphocytes. Then, the SAA mRNA expression was analysed and we observed more SAA transcripts during lactation than gestation.

**Conclusion:**

These data are consistent with the SAA_23–45 _fragment being involved in preplasma B-cell recruitment to the mammary gland and resultant benefit to the neonate.

## Background

The mammary gland (MG) secretion is essential for the survival of neonatal mammals [1]. This organ is a tertiary extralymphoid tissue with non-organised lymphoid components [1,2]. Its vasculature and epithelium expand in size during gestation and lactation. In all mammalian species, this gland is colonised by T and B lymphocytes [3-6]. T lymphocytes accumulate in the MG during gestation whereas B cells and their plasma cell derivatives progressively accumulate during lactation [3,6-8]. Colostrum and milk immunoglobulins (Igs) are the net result of Ig extravasation from blood and local Ig production by plasma cells [9]. These plasma cells are derived from remote tissue precursors which are induced either in systemic and in mucosal sites, depending on Ig isotype. In single-stomached animals, IgA is secreted by IgA plasmablasts, which originate in the gut [6,8,10-12] whereas IgG is derived from systemic sites [13].

It has been shown that the CC chemokine CCL28 [14-16] plays a major role in IgA plasma cell recruitment. Other factors may also be involved, particularly for other types of plasma cell. Indeed, unidentified factors in milk increase the migration of mouse IgG- and IgA-bearing mucosal lymphocytes [17]. For example, tumour necrosis factor isolated from milk has been shown to increase the migration of lymphocytes [18]. Moreover, other studies have reported that milk contains factors with cell regulatory and stimulatory properties. These factors include hormones and cytokines, which do not have specific effects on any given lymphocyte subset [19]. We have shown that swine whey has chemoattractant activity (CA) for swine lymphocytes [20]. We therefore hypothesized that whey was involved in the local recruitment of plasma cells [6,21]. We tested this hypothesis by fractionating sow milk and assessing the ability of purified fractions to increase lymphocyte or B lymphoblast migration in relation to the stage of development of the healthy mammary gland.

## Results

### Chemoattractant activity of whey ultrafiltrate

**Figure 1:**
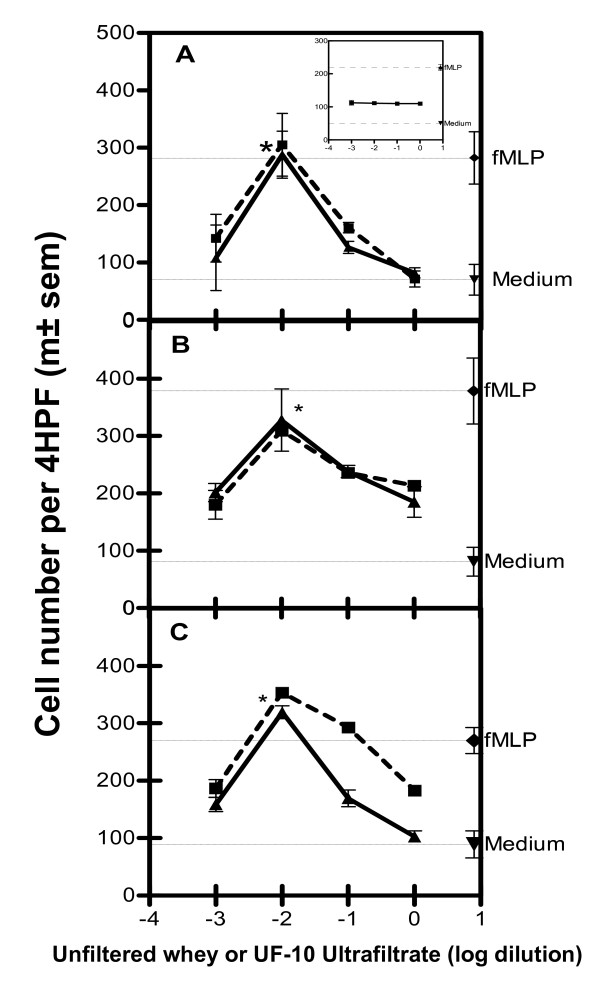
**Chemoattractant activity of whey with lymphoid cells, before (▲ – ▲) and after 10 kDa PES membrane ultrafiltration (■ – ■)**. Swine B-cell lymphoblasts (A), lymphocytes from the MLN (B) and from the ILN (C). Spontaneous and fMLP (10^-6 ^M)-induced migrations are indicated by horizontal lines. In the insert: Absence of chemotactic activity of ultra-retentate onto B lymphoblasts. The results are expressed as the mean number of adherent cells per 4 HPF in 3 independent experiments (except with ILN (n = 6)), each performed in triplicate (*p < 0.05 versus spontaneous migration).

Previous investigations have shown that the removal of fat and casein did not result in the loss of the original CA of skimmed milk (data not shown). We show here that the < 10 kDa ultrafiltrate fraction (UF10) gave a bell-shaped dose-response curve typical of chemoattractants at the same optimal dilution (1/100), and with a similar number of migrating cells (Figure 1A to 1C) to unfiltered whey. On the contrary, the > 10 kDa fraction was inactive (Figure 1A). Lymph node (LN) lymphocytes (Figure 1B and 1C) and B lymphoblasts (Figure 1A) migrated to similar extents. Neutrophils [22] and monocytes (data not shown) purified from the swine blood do not exhibit any CA with fMLP and the < 10 kDa ultrafiltrate fraction. These results combined with morphological data demonstrate that CA activity is restricted to lymphoid cells. As CA was similar for lymphocytes from the mesenteric lymp node (MLN) and the ileal lymph node (ILN), we subsequently evaluated CA principally on B-cell lymphoblasts and MLN lymphocytes.

### RP-HPLC fractionation and purification of the milk chemoattractant

**Figure 2:**
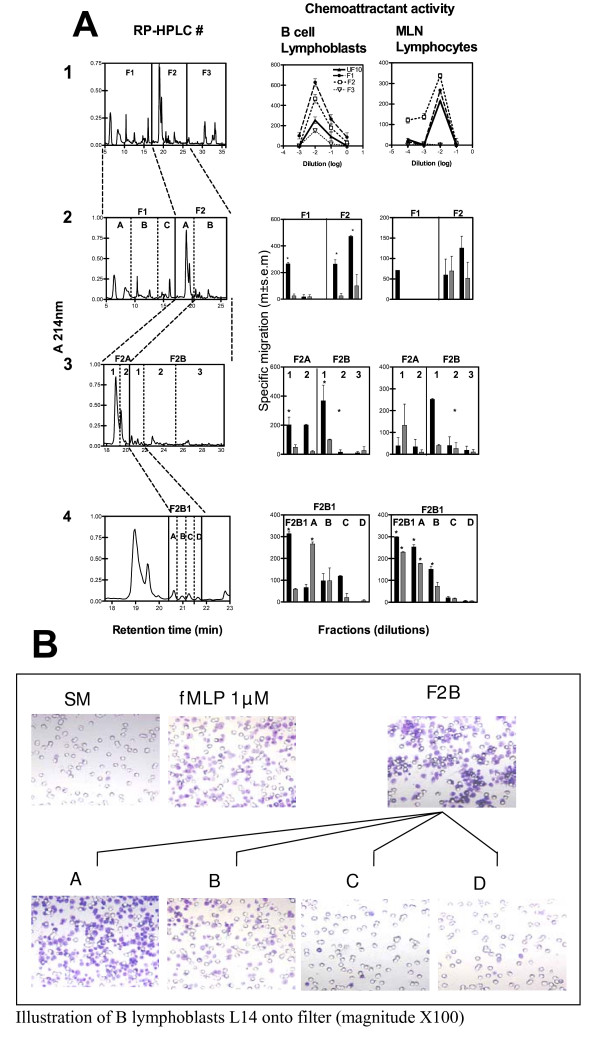
**Subfractionation of < 10 kDa whey ultrafiltrate by successive RP-HPLC (A, left panel 1 to 4), showing specific chemoattractant activity (A, medium and right panels) with B-cell lymphoblasts and MLN lymphocytes**. Each fraction was collected and freeze-dried to remove any residual solvent. Chemoattractant activity was determined at 1/100 (black) and at 1/1000 (shaded) dilutions of the F1 and F2 fractions. The results are the mean number (± SEM) of specific migrating cells in independent experiments (* p < 0.05 versus spontaneous migration). Lymphoblast migration towards 2B1 fraction and subfractions at a dilution of 1/1000 (**B**), in the Boyden's chamber assay (Magnitude ×100). F2B1-A fraction and positive control (fMLP) induced potent chemotaxis. Nearly only the pores (8 μM) in the filter are seen in spontaneous migration (SM) and in F2B1-D fraction.

**Figure 3:**
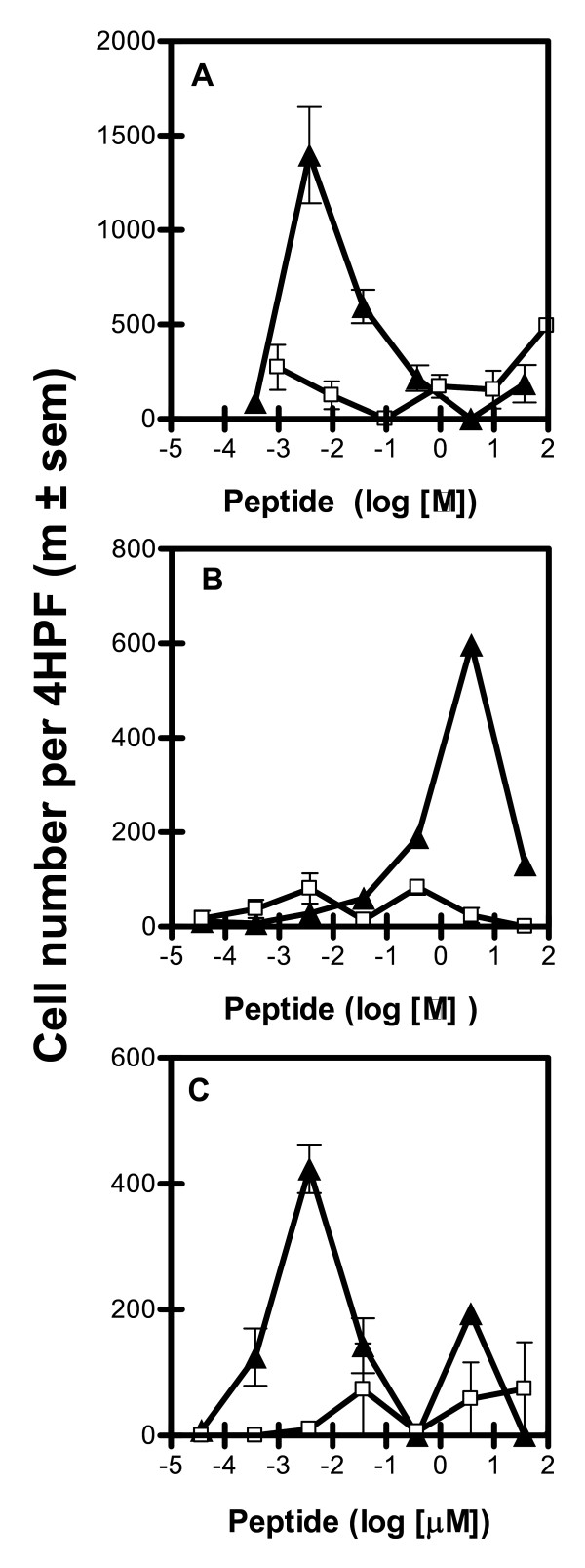
**Chemoattractant activity of 1042 Da (□) and 2735 Da (▲) synthetic peptides with B-cell lymphoblasts (3A); lymphocytes from MLN (3B); and from ILN (3C)**. The results are expressed as the mean number (± SEM) of specific migrating cells per 4 HPF in independent experiments (A, n = 1; B, n = 4; C, n = 2).

**Figure 4:**
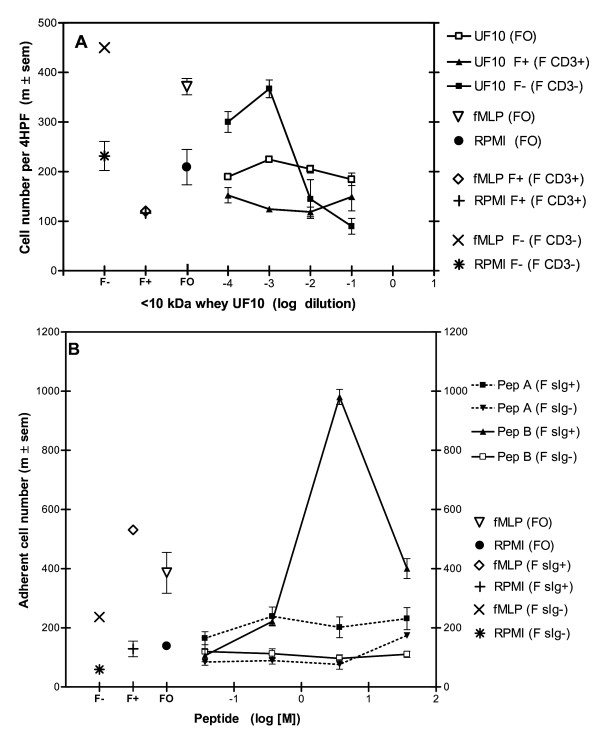
**Chemoattractant activity of < 10 kDa fraction (A) and of synthetic peptides (B) with MLN lymphocytes enriched in CD3 or depleted in SIg+ B cells (at a concentration of 3.7 μM)**. **A) **CD3+ (F+), CD3- (F-) and origin cell (FO) fractions with the < 10 kDa fraction of whey (UF10) and corresponding spontaneous migration (fMLP 10^-6 ^M) and RPMI controls with FO, F+ and F-). **B) **Chemoattractant activity of 1042 Da (PepA) or 2735 Da (PepB) synthetic peptide with the SIg+ B cell-enriched fraction (F sIg+) and chemoattractant activity of the same 1042 Da (PepA) and 2735 Da (PepB) peptides with the fraction depleted of SIg+ B cells (F sIg-). Corresponding spontaneous migration (fMLP 10^-6 ^M and RPMI controls). Results are the mean number of adherent cells ± SEM per 4 high-power fields (HPF).

In two fractions (F1 and F2) of the first RP-HPLC fractionation of UF10 (Figure 2A-1), CA was recovered at the original dilution (1/100), with B lymphoblasts and MLN lymphocytes, whereas F3 was inactive. After further fractionation, only the F2A and F2B fractions were active (Figure 2A-2) with both B-cell lymphoblasts and lymphocytes at optimal and suboptimal dilutions, whereas the F1A fraction was only active at optimal dilution. We fractionated F2A and F2B further and found that only the F2B1 subfraction was active with both B-cell lymphoblasts and MLN lymphocytes at optimal and suboptimal concentrations, whereas the F2A1 and F2A2 subfractions were not active at suboptimal concentrations. The initial CA of UF10 was recovered mostly in the F2B1 subfraction, which was subjected to a fourth run of RP-HPLC, (Figure 2A-4). CA with both lymphoblasts (Figure 3) and lymphocytes was recovered in the F2B1-A and F2B1-B peaks.

### Identification of the chemotactic peptide

The two most chemoattractant peaks, F2B1-A and F2B1-B, were characterised by MS (Table 1). These peaks were found to correspond to five peptides with overlapping sequences. A 3471 Da peptide was found in both the A and B peaks, whereas the 1042 Da peptide was present in peak A but not in peak B. The peptides were sequenced by MS-MS and homology searches of databases (Swissprot) indicated that all sequenced peptides in peaks A and B were derived from the same serum amyloid A protein (SAA), with the exception of an unidentified 3471 Da peptide.

**Table 1:** Tandem mass spectrometry sequences of A and B peaks peptides of the 2B1 fraction obtained from the < 10 kDa fraction after 4 RP-HPLC runs. ^1 ^In italic, sequences of synthetic peptides.

**AA sequence**	**Theoretical mass (Da)**	**Measured mass (Da)**
**Peak A**		

*DMREANYKNSDKYFHARGNYDAA*^1^	2735.21	2734.55
*RPPGLPDKY*	1041.56	1041.30
SDMREANYKNSDKYFHARGNYDAA	2822.25	2821.57
Not identified		3470.92
DMREANYKNSDKYFHARGNYDA	2664.18	2663.52

**Peak B**		

DMREANYKNSDKYFHA	1987.88	1987.82
YSDMREANYKNSDKYFHARGNYDAA	2985.31	2985.27
Not identified		3471.77
DMREANYKNSDKYFHARGNYDAA	2735.21	2735.18
YSDMREANYKNSDKYFHARGNYDA	2914.27	2914.26

We assessed if these peptides derived from SAA were responsible for the CA of the F2B1 fraction, by testing the CA of the synthetic peptides DMREANYKNSDKYFHARGNYDAA (Mr 2735) and RPPGLPDKY (Mr 1042) separately. These two synthetic peptides eluted with the same retention times as peaks A and B from F2B1 in RP-HPLC (Figure 2A-4) under the same conditions (data not shown). We also showed that F2B1-A induced potent chemotaxis (Figure 2B).

The dilution-effect curve (Figure 3A) showed that CA was recovered with the 2.7 kDa peptide, albeit at 1000 times lower concentrations for lymphoblasts (Figure 3B) than for MLN lymphocytes (Figure 3B), whereas the 1.0 kDa peptide had no activity. Furthermore, there were two peaks of CA with ILN lymphocytes (Figure 3C) at 3.7 μM and 3.7 nM. At the lower concentration, most of the migrating cells were lymphoblasts, in clusters of 10 to 25 cells. This result is consistent with the effects on L14 B lymphoblasts, which was observed at the same concentration (Figure 3A). At the higher concentration, the 2.7 kDa peptide induced the migration of small ILN lymphocytes, consistent with the observed effects on small MLN lymphocytes (Figure 3B).

We investigated whether peptides were chemotactic or chemokinetic, using the checkerboard assay (Table 2) with B lymphoblasts. Maximal migration occurred when higher molar concentrations of the 2.7 kDa peptides were placed in the lower compartment and no significant increase in migration was observed when equal molar concentrations of 2.7 kDa peptides were placed on both sides of the filter, thereby demonstrating that the 2.7 kDa residue peptide was not chemokinetic. The 1 kDa peptide did not display any activity potentially attributable to a chemokinetic or chemotactic response.

**Table 2:** Checkerboard assay of lymphoblast migration^a ^towards 2.7 and 1 kDa peptides.

	**Concentration of the 2.7 kDa peptide (nM) above the filter**			
	
**Concentration of the 2.7 kDa peptide (nM) below the filter**	**0**	**3.7**	**37**	**370**
0	100.3^b ^± 11.2	148.6 ± 14.2^c^	77.6 ± 1.7^c^	136.0 ± 9.8^c^
3.7	224.3 ± 2.0^c^	132.0 ± 8.0	114.7 ± 0.3^c^	156.5 ± 7.5
37	228.0 ± 12.7^c^	125.5 ± 13.5	146.0 ± 11.0	125.0 ± 12.0
370	119.0 ± 8.7	87.6 ± 3.2^c^	110.5 ± 0.5	74.3 ± 4.1

	**Concentration of the 1 kDa peptide (nM) above the filter**			
	
**Concentration of the 1 kDa peptide (nM) below the filter**	**0**	**3.7**	**37**	**370**

0	103^b^.0 ± 5.2	103.3 ± 16.8^c^	124.7 ± 8.9	124.6 ± 9.5
3.7	49.0 ± 4.0	70.7 ± 4.2	85 ± 13.3	121.3 ± 39.6
37	64.0 ± 43.1	66.3 ± 8.9	60.7 ± 3.2	147.3 ± 6.8
370	64.3 ± 6.1^c^	49.0 ± 2.9^c^	74.7 ± 11.6^c^	103.3 ± 4.41

^a^Migration was assessed with B lymphoblasts after 1 h of incubation in the presence of peptides in the upper, lower or both compartments. For underlined values, there was no gradient of peptide concentration between the upper and the lower compartments. In each column, above the underlined values, the peptide concentrations were lower than in the upper compartment and below the underlined values, the peptide concentrations were higher than in the upper compartment. Results are means ± SEM of migrating cells, for experiments carried out in triplicate. ^b^Spontaneous migration in the absence of peptide. ^c^Significant difference between migration in the absence of a gradient (underlined values) and peptide-induced migration (p < 0.05 by Student's *t *test). A polycarbonate filter with 12 μm pores separated the two compartments of the chamber.

### Chemoattractant activity with separated CD3+ and sIg+ cells from mesenteric lymph node

CD3+ T lymphocytes and sIg+ B cells were purified by immunomagnetic positive sorting. The enrichment for CD3 was > 95% and the depletion for sIg > 95% (data not shown). The migration of CD3+ T cells in response to UF10 did not differ from spontaneous migration (Figure 4A), whereas the CD3- fraction induced a dose-dependent increase in migration. Conversely, the extent of migration of the purified sIg+ B cells (Figure 4B) towards the 2735 Da peptide was higher than that of spontaneous migration, at a concentration of 3.7 μM (p < 0.01), whereas the extent of migration of the sIg- fraction did not differ (p > 0.05) from that of spontaneous migration. No increase in migration towards the 1042 Da peptide was (p > 0.05) observed in either sIg+ B cells or sIg-depleted cells. Consequently, the 2735 Da peptide is chemoattractant for swine sIg+ B cells, but not for CD3+ T cells. Furthermore, the peptide was not chemoattractant for purified porcine neutrophils and monocytes (data not shown).

### SAA mRNA production in the porcine mammary gland

**Figure 5:**
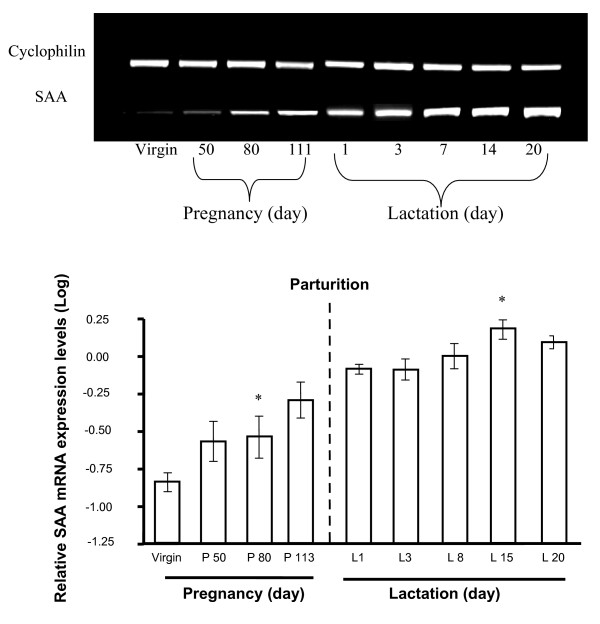
**Expression of SAA in the mammary gland during gestation and lactation, as determined by semi-quantitative reverse transcription-polymerase chain reaction (RT-PCR)**. Upper panel: Profiles of bands obtained for SAA and cyclophilin in porcine MG. Lower panel: Kinetics of SAA mRNA production from the virgin state through gestation and lactation in porcine MG. The relative signal intensities were obtained by normalization with respect to the cyclophilin signal (mean ± SEM of 2–3 animals). (*) Levels of SAA mRNA did not vary during gestation or lactation, until day-80 of gestation, after which they increased significantly, reaching a peak on day 15 of lactation L15: p < 0.05 in Student's *t *tests.

**Figure 6:**
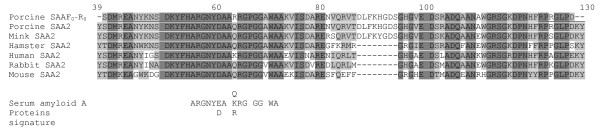
**Comparison of the SAA sequence recovered from the porcine mammary gland (Porcine SAAF0-R0) with sequences of SAA2 isoforms**. Sequences were obtained from the NCBI website , for swine (ABC96790), mink (P02739), hamster (P20727), human (AAH20795), rabbit (P22000) and mouse (AAH24606). Amino-acid position is indicated at the top of the figure and corresponds to the amino-acid number in the Porcine SAA2 sequence (ABC96790). Identical and conservative residues are highlighted in dark and light grey, respectively. Gaps are denoted by a dash. The sequences of the peptides obtained from whey and synthesised in this study are underlined.

We then assessed porcine SAA mRNA expression in the MG as a function of developmental stage. MG cDNA was amplified by PCR using primers (F0 and R0) designed into two relatively well conserved SAA regions. A single PCR product, corresponding to an internal fragment of the porcine SAA cDNA, was obtained (Figure 5). Its deduced amino-acid sequence carried the signature of all serum amyloid A proteins: ARGNYD(E)A-K(Q, R)RG-GG-WA. Moreover, this sequence showed a very high percentage of amino acids identical to those of the SAA2 sequences of *Sus scrofa *[GenBank:ABC96790] now available (Figure 6). As a single sequence was amplified by PCR, this "SAA2" is probably the most predominant member of the SAA family expressed in the MG. Production of the porcine SAA mRNA in MG was then assessed, using a second primer pair (F1 and R1) designed on the basis of our porcine SAA cDNA sequence. In porcine MG, SAA mRNA was detectable during gestation, and its level began to increase significantly at day 80 of gestation, reaching a peak at day 15 of lactation (Figure 5). The SAA mRNA expression was higher in the second half of lactation than at any stage of gestation (p < 0.05).

### Peptide-induced mobilization of intracellular Ca^++ ^in differentiated HL-60 cells and FPRL2-expressing HL-60 cells

**Figure 7:**
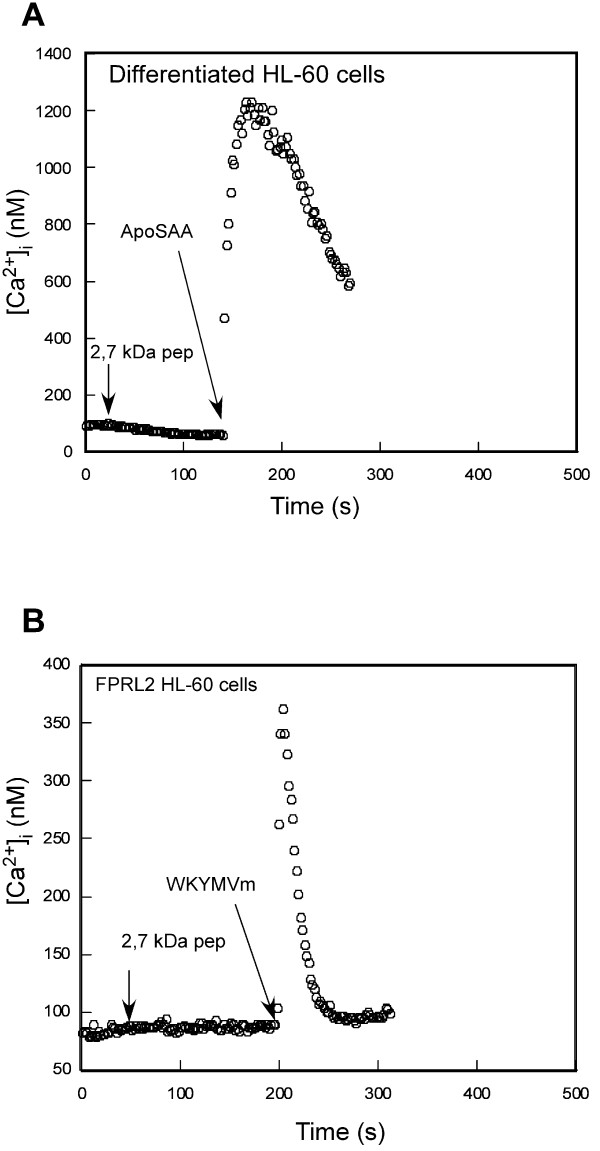
**Calcium mobilization in myeloid cells expressing FPR, FPRL1 or FPRL2**. Differentiated HL-60 cells and undifferentiated FPRL2-expressing HL-60 cells were loaded with 2 μM Fura-2 and analyzed with respect to calcium mobilization mediated by the addition of peptides. (**A**) Addition of the 2,7 kDa fragment from SAA2 (1 μM) to differentiated HL-60 cells was unable to mobilize intracellular calcium whereas 100 nM of recombinant of human ApoSAA, an agonist of FPRL1, induced a robust calcium rise (**B**) Undifferentiated HL-60 cells that stably express FPRL2 were unable to mobilize intracellular calcium in response to the addition of 1 μM of the 2,7 kDa fragment from SAA2 whereas a calcium rise was observed upon addition of 100 nM of WKYMVm, an agonist of FPRL2.

Because the 2.7 kDa peptide (DMREANYKNSDKYFHARGNYDAA) exhibits a high degree of amino acid identity with human SAA, which previously has been shown to bind to and to activate the human N-formyl peptide receptor-like 1 (FPRL1), we tested whether it could activate a FPRL1- or FPRL2-dependent calcium response. FPRL1 is a member of the FPR family that is expressed in human neutrophils and differentiated HL-60 cells as well as monocytes, whereas the N-formyl peptide receptor-like 2 (FPRL2) is expressed in human monocytes but not in neutrophils. When added to differentiated HL-60 cells or undifferentiated FPRL2-expressing HL-60 cells, the 2.7 kDa synthetic peptide (DMREANYKNSDKYFHARGNYDAA) was unable to induce intracellular calcium mobilization. In contrast, recombinant human ApoSAA, an agonist specific of FPRL1, or WKYMVm, an agonist of FPRL2, triggered a robust calcium response in differentiated HL-60 cells or undifferentiated FPRL2-expressing cells, respectively. This observation indicates that the absence of response with B cells could not be due to a lack of expression of FPRL1 (Figure 7A) or FPRL2 (Figure 7B).

## Discussion

In this study, most of the CA of milk for B lymphocytes and B lymphoblasts was detected with the 2735 Da peptide isolated from the whey while no CA activity was detected in the 1042 Da peptide from the same fraction. CA activity was not detected for neutrophils and monocytes, either onto filter or with purified blood cells. Amino-acid sequencing of the two peptides showed that they belonged to the SAA protein family [23]. The partial sequence of the SAA cDNA amplified from the MG suggested that these peptides might be derived from SAA2, as the DNA sequence obtained was 98% identical to that of porcine SAA2 [24]. However, we cannot draw definitive conclusions concerning the SAA subtype corresponding to the 2.7 kDA chemoattractant peptide, as this peptide displayed a high degree of identity (> 90%) with SAA subtypes from different origins. Our data suggesting that SAA2 predominates in the MG, are reminiscent of the finding that bovine mammary-SAA or SAA3 differs from serum-AA in being present at extrahepatic sites, such as the gut epithelium, MG epithelium [25] and in colostrum and milk in healthy animals [26]. However, human breast was found to contain SAA1, SAA2 and SAA4, indicating that there is considerable variation amongst species, despite the sequence similarities. In swine SAA2, there is no deletion of the 8 aa (number 88 to 95) of the human sequence required for acute inflammation reaction [27]. This observation provides further evidence that this peptide could play a physiological role in normal MG. The catabolic pathway of SAA has yet to be completely defined, but various studies have suggested that SAA is degraded by cell-associated elastase-like proteases [28,29] and several other serine proteases found in serum and milk, including kallikrein and plasmin [30-32]. The proteolysis of SAA by these enzymes leads to the generation of specific peptide fragments, some corresponding to amino acids 29 to 42 of SAA sequence, whereas the chemoattractant peptide is longer (23–45) and has different cleavage sites. The specific action of the mammary SAA peptide on B lymphoid cells contrasts with the previously described activity of SAA. Indeed human SAA is described as involved in adhesion induction, migration, and tissue infiltration of T lymphocytes [33]. The 2.7 kDa peptide (DMREANYKNSDKYFHARGNYDAA) is 92.3% identical to the 29–42 peptide of hSAA-A (YIGSDKYFHARGNY) [34], suggesting a similar function. However, only a few studies have used synthetic peptides: One study reported that hSAA_29–42 _inhibited adhesion [34], whereas another found no inhibitory effect on neutrophil migration [35].

It has been shown that hSAA and the 29–42 fragment display agonist activity involving FPRL1 receptor [36,37]. Moreover, fMLP receptor has not only been described on human neutrophils and NK cells but also on B cells [38,39]. As in our study, the fact that B cells respond to μmolar concentrations suggest that they exhibit FPRL1 or FPRL2 receptors. However, we may exclude FPRL2 as this receptor does not bind or respond to N-formyl peptides [38]. On the other hand, swine L14 B cells incubated with human apoSAA or swine 2.7 kDa peptide failed to exhibit any calcium flux. These results indicate that the peptide probably uses a receptor other than FPRL1 and FPRL2. This receptor could be the recently described "receptor independent of FPRL1 [40], which did not lead to a calcium influx. Furthermore, this receptor could be present in two similar forms: One of lower avidity on B cells and the other of higher avidity or subject to up-regulation after the activation of B cells or B lymphoblasts. Alternatively, these results could fit with the observation that B lymphocytes can respond without mobilization of intracellular calcium as observed, for example, with chemokines CCL19, CCL21, CXCL12 and CXCL13 [41]. The lack of CA of the terminal SAA peptide (RPPGLPDKY) with B cells does not entirely rule out activity with these cells. Indeed, the truncated form of hSAA, AGLPDKY (SAA_98–104_), and analogues are known to bind human CD4+ T lymphocytes and to stimulate them to produce interferon-γ. It is also possible that the 98–104 peptide makes the cells susceptible to the 29–42 fragment chemoattraction after activation.

Binding may also lead to the induction of cellular responses, such as cytokine secretion [42] and the up-regulation of integrin α4β1, in particular. α4β1 expression increases the probability of *in vivo *binding to VCAM-1 [43], a vascular addressin present on the MG blood vessels [6]. The lymphocyte aggregation observed in the present study (Figure 2B) would be consistent with the expected effect of such binding.

The YIGSD laminin-like element of hSAA is replaced by the YKNSD element in pSAA, and both hSAA and pSAA express RGN motif, a "RGD like" motif. This RGN motif [34] represents integrin-binding and cell attachment motifs derived from cellular adhesion protein molecules, such as fibronectin, collagen, vitronectin, fibrinogen, laminin and many microbial proteins, including the Tat protein of the human immunodeficiency virus [44,45]. Binding of the RGN motif to the cell membrane is known to stimulate membrane integrins, leading to a local increase in the concentrations of F-actin and α-actinin, resulting in cell locomotion [46]. It is possible that the peptide adheres to the filter via this RGN motif, inducing lymphocyte migration. Transposed *in vivo*, these data suggest a similar phenomenon, in which the binding of SAA to the surface of the mammary endothelial cells may induce B-cell adhesion followed by the transendothelial migration of these cells in an SAA-peptide gradient. Finally, in addition to binding to endothelial cells, SAA also binds to the basal membrane and extracellular matrix, probably due to its heparin-binding properties [47]. Thus, gradients of extracellular tissue-bound SAA or SAA peptides, including the 2.7 kDa peptide, may induce haptotactic B-cell migration in the mammary tissue during lactation.

SAA mRNA and protein productions have been recently demonstrated in extrahepatic tissues, and specifically in MG, mostly in epithelial and endothelial cells [25], supporting the hypothesis of a role in lymphocyte recruitment. High extrahepatic expressions and the secretion of the mammary-associated serum amyloid A 3 isoform (M-SAA3) in the colostrum and milk of healthy animals has been reported [26]. Although the functional significance of extrahepatic SAA production remains unclear, it has been suggested that the apparent constitutive production of the SAA transcript in non-hepatic cell lines is consistent with a housekeeping role for the protein [48], either as a first line of defence or in the maintenance of tissue function.

Previous findings of the upregulation of SAA gene expression in response to prolactin [49] and the increase in B lymphoblast numbers in the MG during lactation [6,21] would together be consistent with the results of the present study. The human MG produces and releases bioactive prolactin *in situ *[50]. In this study, we showed that porcine SAA mRNA production increased during lactation, consistent with the hormonal regulation of SAA production mediated by an increase in prolactin concentration at this stage of MG development. This milk peptide, due to its small size, could cross the neonatal gut epithelium and stimulate the neonatal gut immune response by enhancing the recruitment of mucosal gut B lymphoblasts.

## Conclusion

In the current paper, we have investigated whether physiological chemoattractants induced the recruitment of immune cells from the maternal bloodstream in lactating mammary glands, by analysing the chemoattractant activity of various milk fractions. The B cell chemoattractant activity of a SAA 2.7 kDa peptide and the concomitant up-regulation of SAA mRNA during lactation are consistent with the SAA_23–45 _fragment being involved in preplasma B-cell recruitment to the mammary gland, potentially influencing Ig concentrations, conferring active and passive protection on neonates and providing local protection for the mammary gland.

## Methods

### Cell isolation and culture

Cryopreserved cells from the swine L14 lymphoblastoid cell line (ECACC number 91012317) [51] were cultured and harvested in the exponential growth phase, as previously described [20,52]. L14 cells is a permanent swine lymphoblastoïd cell line which has been cloned and confirmed to be of B cell lineage [51-54]. In contrast to monocytes, neutrophils, T cells, NK cells and null cells the L14 cells are sWC1- as the B cells [55]. In addition they are Ig light-chain λ+ (46%), IgM+ (37.5% to 82%), IgA-, IgG-, cytoplasmic light-chain+ (10%), cytoplasmic IgM+ (7%), Ig CD1- [51], CD5- (B2 lymphocytes), CD45+, CD45RA+ (Naïves, 40%), CD45RC+ (35 to 80%), CD29+ (β1, 50% to 80%), CD62L+ (15%), CD11b-, CD11c-, Fc gamma-, CD40+ (100%, responsible of isotopic commutation), CD79b+ (pre-B-cells and mature-B-cells, 40%), CD18-, CD21-, CD49d-, CD49e+ (100%), α4β7-, class II+ (DR and DQ, 99%). Furthermore, L14 B cells are chemoattracted by CCL28 and expressed the corresponding receptor, CCR10 [13].

Lymphocytes were isolated from mini-pig (SLA^d/d^) MLN and ILN and cryopreserved as previously described [56]. Immediately before the test, the cells were cultured overnight at 37°C in a six-well tissue culture plate (Costar, Bethesda, MD, USA), in the presence of 5% CO_2_, at a density of 10^6 ^cells/ml, in RPMI 1640 (Gibco, Cergy-Pontoise, France) supplemented with 2 mM L-glutamine (Sigma-Aldrich, Saint-Quentin, France), 1 mM pyruvate (Sigma-Aldrich, Saint-Quentin, France), and 50 μM β-mercaptoethanol (Sigma-Aldrich, Saint-Quentin, France), to obtain cells with a membrane integrity > 95%, as determined by Trypan blue exclusion. At the end of the incubation period, both plastic-adherent and non-adherent cells were collected. The dead cells were eliminated by gradient centrifugation (d = 1.09) and the viable cells were suspended in RPMI 1640 medium (Gibco, Cergy-Pontoise, France) without foetal bovine serum for chemoattractant assays.

### Magnetic cell sorting of CD3+ and sIg+ B lymphocytes

We resuspended 200 × 10^6 ^MLN lymphocytes in PBS without Ca^2+ ^or Mg^2+^, and incubated them with swine serum for 5 min at room temperature to block the Fc receptors. They were then washed twice in PBS without Ca^2+ ^and Mg^2+^. Cells were incubated on a shaker for 30 min at 4°C with monoclonal mouse anti-CD3 (BB238 E6) or anti-swine Ig λ light chain antibody [57]. We then added 200 μl of anti-mouse immunoglobulin magnetic microbeads (Miltenyl Biotec, Aurburn, CA, USA) to 10^8 ^cells in 800 μl of buffer and the mixture was incubated on a shaker for 30 min at 4°C. The cells were then washed twice with PBS without Ca^2+ ^or Mg^2+ ^and resuspended in 500 μl of the same buffer. The cells and beads were loaded onto columns (LST, Miltenyl Biotec, Auburn, CA, USA), which were placed on a MidiMACS separator. The cells not bound to the magnetic beads flowed through readily. The columns were washed with MACS medium (PBS without Ca^2+ ^or Mg^2+ ^supplemented with 1 mg/ml bovine serum albumin and 2 mM EDTA). Then, the columns were removed from the separation unit and filled with MACS media. A plunger was inserted into the columns to flush out the CD3+ T cells or Ig light chain-positive cells. The purity of the positive and negative cells was assessed by FACS analysis.

### Flow cytometry analysis

Cell suspensions in V-bottomed microtitre plates were immunostained as follow: 5 × 10^5 ^cells per well were washed with 200 μl flow cytometry medium (FCM, 1% BSA and 0.1% sodium azide in PBS) and incubated with 50 μl goat serum for 5 min at room temperature, to block non-specific binding. Cells were washed three times in FCM and incubated for 30 min with 50 μl of mouse anti-CD3 antibody or mouse anti-swine Ig λ light chain antibody with gentle stirring at 4°C in the dark. Then, they were washed and incubated with 50 μl of goat anti-mouse IgG RPE (DAKO R 0480, Glostrup, Denmark). Finally, cells were resuspended in 100 μl FCM and analysed on a Becton-Dickinson FACScan [58,59].

### Chemotaxis assay

Cell chemoattraction was measured in 48-well microtaxis chambers (Neuroprobe, Cabin John, MD, USA) as previously described [56]. Briefly, the lower compartment was filled with 28 μl of chemoattractant solution, using fMLP 10^-6 ^M (Sigma-Aldrich F-3506, lot 48f5805, Saint-Quentin, France) as the positive and medium only (RPMI 1640 without FCS) as the negative control. It has been shown previously that fMLP is not chemoattractant in swine for neutrophils [22] but for lymphocytes [56]. Moreover, in human, fMLP chemoattracted specifically B cells but not T lymphocytes [60] and recently its receptor has been found onto B cells and NK cells [39]. We then transferred 48 μl of cell suspension at a density of 4 × 10^6^cells/ml to the upper compartment and 25 μl of medium with or without CA were placed in the lower compartment. The upper and lower compartments were separated by a polycarbonate PVP-free membrane with 8 μm or 12 μm pores for lymphocytes and lymphoblasts, respectively. Chambers were incubated for 1 h at 37°C and the filter was then removed and stained with haematoxylin [56]. Migration was assessed by counting the number of lymphocytes that had migrated through the pores and adhered to the lower surface of the filter in four randomly chosen high-power fields, using a 40× oil objective and a 10× eyepiece with a Sony 3CCD colour video camera (Sony, Olympus, Japan) and AnalySIS software (Soft Imaging System, Olympus, Japan). All samples were tested in triplicate. Specific migration was evaluated in some experiments.

We investigated whether synthetic peptides of SAA were chemokinetic (enhanced random migration) or chemotactic (directed migration) by carrying out a checkerboard assay as previously described [61].

### Milk sampling

Milk was obtained five days into lactation, from healthy control animals at the INRA farm (St Gilles, France). No evidence of clinical mastitis was found at the time of sample collection or during the two-month follow-up period. The milk was skimmed by centrifugation at 2000 *g *for 15 min at 4°C and caseins were removed by ultracentrifugation at 85000 *g *for 1 h at 4°C. The separated fat, whey and casein fractions were stored at -20°C until further analysis. Our previous data show that these processes do not result in the loss of the original CA of the skimmed milk.

### Ultrafiltration of whey

Whey proteins were subjected to ultrafiltration through centrifugal concentrators (Vivaspin 20, Sartorius AG, Goettingen, Germany) at 3000 *g *and at room temperature, using a 30 kDa polyethersulfone membrane (UF30) and then a 10 kDa polyethersulfone (UF10) membrane. The ultrafiltrates obtained by separation with membranes of different thresholds were reconstituted to their initial volume with PBS and tested for CA. Salts and milk sugars were eliminated by solid-phase extraction (SPE) with a C18 grafted silica phase. The retained molecules were washed with 0.1% TFA to remove salts and milk sugar and then eluted from the silica with an aqueous acetonitrile solution (80%), and freeze-dried.

### Fractionation of ultrafiltrate

Ultrafiltrate UF10 was fractionated by semi-preparative RP-HPLC on a Vydac C18 column (218TP510, 150 × 10 mm I.D). The column was equilibrated in 100% solvent A (1.06‰ TFA in milliQ water). Ultrafiltrate UF10 (500 μl) was injected and after 5 min at 5 ml/min in isocratic mode, elution was carried out with a linear gradient increasing to 70% solvent B (1.0‰ TFA in 80% aqueous acetonitrile) over 40 min. The column temperature was 40°C and elution was monitored at 214 nm. A sequential subfractionation of UF10 (Figure 2A) was carried out to isolate the chemoattractant factor, and we focused on the most chemoattractant fraction at each step. All fractions were freeze-dried and then resuspended in the same volume of RPMI as of sample initially injected in RP-HPLC, for CA evaluation. Three fractions (F1, F2 and F3) were initially obtained. The F1 and F2 fractions were further subdivided, by injection of a second aliquot of UF10, leading to harvesting of the F1A, F1B, F1C, F2A and F2B subfractions. As CA was highest in the F2A and F2B fractions, these fractions were further subfractionated to give F2A1, F2A2, F2B1, F2B2 and F2B3. In the last step of RP-HPLC purification, the F2B1 fraction was further fractionated to give F2B1- A, B, C and D.

### Mass spectrometry and tandem mass spectrometry analysis of chemoattractant factor

Mass spectrometry measurements were performed at the research support facility "Plant Biopolymers – Interactions – Structural Biology", INRA, at Nantes. Mass data analyses were conducted on a hybrid quadrupole orthogonal acceleration time-of-flight mass spectrometer (Q-TOF Global, Waters, Guyancourt, France) equipped with a nano-flow ESI interface.

For mass analyses, samples were dissolved in 8 μl of a water:acetonitrile:formic acid mixture (49.5:49.5:1, v/v). Approximately 1 μl of sample was introduced into a nano-ESI precoated borosilicate capillary (MDS Proteomics Inc., Odense, Denmark) and infused into the ion source at a flow rate of about 50 nl/min. MS and MS/MS measurements were carried out under the control of Mass Lynx software (Waters, Guyancourt, France).

### Synthetic peptides

Two peptides were synthesized at Neosystem SA (Neosystem SA, Strasbourg, France), with the amino-acid sequences RPPGLPDKY (Mr 1041.56 Da) and DMREANYKNSDKYFHARGNYDAA (Mr 2735.21 Da). Purity exceeded 98%, as determined by high performance liquid chromatography (Neosystem SA, Strasbourg, France).

### RNA extraction and DNase treatment

Samples of porcine MG tissue (0.1–0.2 g) were collected into 1 ml Trizol (Invitrogen, Cergy Pontoise, France) in polypropylene tubes and total RNA was extracted according to the manufacturer's recommendations. After isopropanol precipitation, the RNA pellet was washed with 75% ethanol, dried and resuspended in 84 μl of ultrapure water containing 0.2% (w/vol) diethylpyrocarbonate (Sigma-Aldrich, Saint-Quentin, France). Possible DNA contamination was eliminated by DNase treatment (Promega France, Charbonnières, France) for 30 min at 37°C, in a final volume of 100 μl containing: 4 U RQ1 RNase-free DNase, 40 U RNasin ribonuclease inhibitor (Promega France, Charbonnières, France), 1 × RQ1 DNase buffer (Promega France, Charbonnières, France). We added 1 μl Stop Solution and incubated the mixture at 65°C for 10 min. RNA was quantified by determining absorbance at 260 nm and purity was assessed by determining the A_260_/A_280 _ratio.

### Reverse transcription

Reverse transcription was performed in an iCycler thermocycler (Bio-Rad, Hercules, CA), using 0.2 ml thin-walled tubes (Applied Biosystems, Foster City, CA, USA). RNA (5 μg) was first incubated at 65°C for 10 min with 0.5 μg oligodT_(12–18) _(Invitrogen, Cergy Pontoise, France), and reverse transcription was then initiated by adding 5 U AMV reverse transcriptase (Promega France, Charbonnières, France), 32 U RNasin (Promega France, Charbonnières, France), 1 mM each dNTP (Promega France, Charbonnières, France) and 1× AMV reverse transcriptase buffer, in a final volume of 40 μl. The reaction was allowed to proceed for 1 h at 42°C and cDNA was stored at -20°C.

### PCR amplification of porcine SAA in mammary gland and densitometric quantification

We carried out a BLASTN search of the GenBank dbEST database. As no homologous porcine EST was identified based on the porcine SAA cDNA sequence, primers F0 et R0 were designed from dog, mink, guinea pig, rabbit and human SAA cDNA sequences, CM59173, in a region relatively well conserved between SAA of different subtypes and different animal origins, using VectorNTI software (Invitrogen, Cergy Pontoise, France). The forward degenerate primer was F0 (5'-TCAAGGGGCTTGGGACATG-3') and the reverse degenerate primer was R0 (5'-AGGGCAGAGCCAAGAGGAAG-3'), yielding a PCR product of 330 bp. PCR amplification was performed using the *Pfu *DNA polymerase (Promega France, Charbonnières, France).

PCR was carried out for SAA cDNA amplification, in a total volume of 20 μl containing 100 ng of cDNA, 1 U Taq polymerase (Promega France, Charbonnières, France), 0.2 mM of each dNTP, 0.5 μM of each primers, 1× *Taq *polymerase buffer (Promega France, Charbonnières, France), 2 mM MgCl_2 _(Promega France, Charbonnières, France). Sense F1 (5'- TCTGACATGAGAGAGGCCAACTAC-3') and anti-sense R1 (5'-GTCAGGCAGGCCACGAGGTCT-3') primers were designed on the basis of our porcine SAA cDNA sequence obtained from the F0-R0 PCR product. The porcine cyclophilin gene was used as a constitutively expressed 'housekeeping' gene control, to determine the uniformity of the reverse transcription reactions. Primers were used as previously described [59]. The amplification conditions were carefully chosen to obtain signals in the linear range of amplification. Amplification products (10 μl) were loaded onto a 2% TBE agarose gel stained with ethidium bromide (0.01%), subjected to electrophoresis and visualised under a UV transilluminator. The fluorescence intensity of the bands was determined using the Alpha Imager Gel Analysis System Fluorchem version 2.00 (Alpha Innotech Corporation, San Leandro, CA, USA). A single PCR product, of 270 bp for porcine SAA and 369 bp for cyclophilin, was obtained and analyzed by DNA sequencing.

### Culture and differentiation of HL-60 cells and stable expression of N-formyl peptide receptor-like 2 in undifferentiated HL-60 cells

Promyelocytic HL-60 cells were cultured in RPMI 1640 medium/glutaMax I supplemented with 10% of heat inactivated foetal calf serum. The maximal density was maintained below 2 × 10^6 ^cells/ml and, at each passage, cells were centrifuged and resuspended in an appropriate volume of complete medium in order to reach a density of 2 × 10^5 ^cells per ml. To trigger the expression of both N-formyl peptide receptor (FPR) and N-formyl peptide receptor-like 1, HL-60 cells were differentiated to a neutrophil-like phenotype by adding *N*^6^, *O*-2'-dibutyryl adenosine 3',5' cyclic monophosphate (Bt_2_cAMP) at the final concentration of 1 mM for 3 days. The stable expression of FPRL2 in undifferentiated HL-60 cells has been previously described [62].

### Determination of changes in cytosolic calcium in differentiated HL-60 cells and undifferentiated FPRL2 expressing HL-60 cells

Cells were washed with PBS and the cell pellets were suspended, at a density of 2 × 10^7 ^cells/ml, in RPMI medium without phenol red containing 0.1% BSA. Cells were supplemented with Fura 2-AM (Molecular Probes, Eugene, OR, USA) at a final concentration of 2 μM for 30 min, at 37°C. Cell suspensions were then diluted with two volumes of the same medium without BSA, washed once in 10 ml of Ringer-Krebs buffer, and suspended in RPMI medium without phenol red at a density of 2 × 10^7 ^cells/ml. Calcium measurements were carried out with a SPEX FluoroMAX fluorescence spectrophotometer with excitation wavelengths of 340 and 380 nm (the ratio 340/380 is calculated when the signal is weak), an emission wavelength of 505 nm and slit widths of 5 and 10 nm, respectively. Intracellular free calcium concentrations were calculated using the following formula: (Ca^2+^)_i _= *K*_D_(F-F_min_)/(F_max_-F) with a *K*_D _for Fura-2 of 224 nM; F_max _is the fluorescence in the presence of 0.04% Triton X-100 and F_min _the fluorescence obtained after addition of 5 mM EGTA plus 30 mM Tris-HCl, pH 7.4.

### Expression of results and statistical analysis

The results of migration assays are expressed in each experiment as the mean number of migrating lymphocytes ± SEM of triplicates. Specific migration was calculated as the difference between the mean number of lymphocytes migrating in the presence of chemoattractant and the mean number migrating in the control medium (i.e. undergoing spontaneous migration). SAA mRNA levels are presented as the mean ± SEM of the ratio of the band intensity of the SAA RT-PCR product over that of the corresponding cyclophilin RT-PCR product (Log SAA – Log Cyclophilin). Means were compared by variance analysis and the Dunnett's *t *test or Student's *t *test. Differences were considered significant if p < 0.05. All statistical evaluations were carried out and graphs plotted with GraphPad Prism 4.03 (GraphPad software, Inc., San Diego, CA, USA).

## Abbreviations

CA: Chemoattractant Activity; MG: Mammary Gland; MLN: Mesenteric Lymph Node; ILN: Inguinal Lymph Node; FCM: Flow Cytometry Medium; fMLP: formyl-Methionyl-Leucyl-Phenylalanine; MS: Mass Spectrometry; MS/MS: Tandem Mass Spectrometry.

## Authors' contributions

BJR and CC participated in the study design, performed the experiments, and helped to draft the article. GH, DM, JL performed some analysis (MS-MS, purification), and helped to draft the article. IVP and GH helped to design primers, to carry out PCR analysis, and helped to draft the article. MB helped to carry out PCR, and to draft the manuscript. FB carried out the calcium flux experiments, and helped to analyse the results. FM wrote the article. HS conceived the study, participate in the study design and coordination, and wrote the manuscript. All authors read and approved the final manuscript.
